# Natural history of mental health competence from childhood to adolescence

**DOI:** 10.1136/jech-2021-216761

**Published:** 2021-08-16

**Authors:** Meredith O'Connor, Sarah J Arnup, Fiona Mensah, Craig Olsson, Sharon Goldfeld, Russell M Viner, Steven Hope

**Affiliations:** 1 Murdoch Children's Research Institute, Royal Children's Hospital, Parkville, Victoria, Australia; 2 Department of Paediatrics, The University of Melbourne, Parkville, Victoria, Australia; 3 Centre for Social and Early Emotional Development, School of Psychology, Faculty of Health, Deakin University, Geelong, Victoria, Australia; 4 UCL Great Ormond Street Institute of Child Health, London, UK

**Keywords:** health inequalities, life course epidemiology, mental health

## Abstract

**Background:**

Mental health competence (MHC) involves psychosocial capabilities such as regulating emotions, interacting well with peers and caring for others, and predicts a range of health and social outcomes. This study examines the course of MHC from childhood to adolescence and patterning by gender and disadvantage, in Australian and UK contexts.

**Methods:**

*Data:* Longitudinal Study of Australian Children (n=4983) and the Millennium Cohort Study (n=18 296). *Measures:* A measure capturing key aspects of MHC was derived summing items from the parent-reported Strengths and Difficulties Questionnaire, assessed at 4–5 years, 6–7 years, 10–11 years and 14–15 years. *Analysis:* Proportions of children with high MHC (scores ≥23 of range 8–24) were estimated by age and country. Random-effects models were used to define MHC trajectories according to baseline MHC and change over time. Sociodemographic patterns were described.

**Results:**

The prevalence of high MHC steadily increased from 4 years to 15 years (from 13.6% to 15.8% and 20.6% to 26.2% in Australia and the UK, respectively). Examination of trajectories revealed that pathways of some children diverge from this normative MHC progression. For example, 7% and 9% of children in Australia and the UK, respectively, had a low starting point and decreased further in MHC by mid-adolescence. At all ages, and over time, MHC was lower for boys compared with girls and for children from disadvantaged compared with advantaged family backgrounds.

**Conclusions:**

Approaches to promoting MHC require a sustained focus from the early years through to adolescence, with more intensive approaches likely needed to support disadvantaged groups and boys.

## Introduction

As children grow up, they are challenged to develop a range of complex psychosocial skills appropriate to their age, such as regulating emotions and behaviour, interacting with peers and caring for others.^1 2^ While these positive aspects of mental health have been conceptualised in varying ways, the construct of mental health competence (MHC) emphasises success in meeting the ‘developmental tasks expected of individuals of a given age in a particular cultural and historical context’.^1 3^ By this definition, MHC is multidimensional, socioculturally contextualised and not reducible to the absence of psychological distress.^3–5^

In addition to being a valued outcome in its own right, MHC predicts a broad range of psychosocial, health and educational outcomes,^6 7^ and supports children’s capacity to grow into healthy adults leading productive and meaningful lives.^2 3 8^ Recognising this, interest is growing in the potential to support the development of MHC in the child and adolescent years.^2 7^ For example, meta-analytic evidence has shown that school-based social and emotional learning programmes can achieve sustained benefits in key MHC skill areas.^9^

An understanding of how MHC develops over time has important implications for the design and evaluation of such intervention efforts.^10 11^ Evidence as to how MHC develops with age can inform the design of intervention strategies targeting particular periods of development (eg, the transition to adolescence), while an understanding of how MHC unfolds for individual children can direct attention to particular types of trajectories (eg, declining MHC).^12^ However, the capacity to generate this evidence has been hampered by a lack of measurement tools and paucity of longitudinal population data suitable for the assessment of MHC. Indeed, indicators of MHC for children and adolescents have only been developed for use in epidemiological research in recent years.^5 6^

Findings from cross-sectional analyses suggest differences in MHC by particular population characteristics. Children living in more advantaged neighbourhoods already have higher levels of MHC at 5 years of age compared with their disadvantaged peers,^13^ and maternal education and socioeconomic advantage at the family level are associated with subsequent child MHC.^5^ MHC also differs by sex, with girls showing higher levels of MHC in early and late childhood.^6 13^ There is considerable interest in understanding the development of these differences and potential intervention points as an opportunity to maximise future human capital.^14–16^

Recent advances in the measurement of MHC that capitalise on existing population data^5 6 13^ provide an opportunity to address knowledge gaps and describe the development of MHC over childhood and adolescence. Here, we aim to examine (1) the course of MHC from 4 to 15 years, describing change in the prevalence of high MHC as children grow older and change within individuals over time, and (2) the extent to which MHC differs by gender and socioeconomic circumstances. We draw on aligned data from two large population-based cohort studies in Australia and the UK, allowing us to determine common developmental trends between these different countries and cohort designs.

## Method

### Data sources

#### Longitudinal Study of Australian Children

The Longitudinal Study of Australian Children (LSAC) is a nationally representative sample of two cohorts of Australian children, including a Kindergarten (K) cohort of 4983 4-year-olds.^17^ The study commenced in May 2004; to date, nine waves of data have been collected. The LSAC sample selection is documented elsewhere.^17^ A clustered design based on postcode and drawing on the comprehensive Medicare database was used to select a sample that is broadly representative of all Australian children, with the exception of the small proportion of children living in highly remote geographic areas.^17^ Multiple information sources are used, including parent interview; direct child assessments and observational measures; parent, teacher and self-report questionnaires; and linkage to administrative data sets.

#### Millennium Cohort Study

The Millennium Cohort Study (MCS) is a nationally representative cohort of children born in the UK between September 2000 and January 2002, described elsewhere.^18^ Families were selected through Child Benefit Records, and a disproportionately stratified clustered sampling design was used to over-represent children living in Wales, Scotland and Northern Ireland, disadvantaged areas and, in the case of England, areas with high proportions of ethnic minority groups. In all, 18 818 infants were enrolled in the study, with 18 296 singletons present at 9 months (the analysed sample herein). Interviews were carried out by trained interviewers in the home with the main respondent (usually the mother).

### Cohort measures and alignment

We used four waves of data collection in each cohort that included MHC assessment and aligned well by age at 4–5 years, 6–7 years, 10–11 years and 14–15 years (table 1).

**Table 1:** Analysed waves of the Longitudinal Study of Australian Children (LSAC) and Millennium Cohort Study (MCS)

Cohort	Wave	4–5 years	6–7 years	10–11 years	14–15 years
LSAC K	Wave no.	1	2	4	6
	Year	2004	2006	2010	2014
	N	4983	4464	4169	3537
	Mean age (years)	4.8	6.8	10.8	14.9
	Age range (years)	4–5.6	5.9–7.8	10–11.7	14–15.8
MCS	Wave no.	3	4	5	6
	Year	2006	2008	2012/2013	2015/2016
	N	15 040	13 681	13 112	11 576
	Mean age (years)	5.2	7.2	11.2	14.3
	Age range (years)	4.4–6.1	6.3–8.1	10.2–14	13.1–15.3

K, Kindergarten cohort.

#### MHC from 4 to 15 years

The Strengths and Difficulties Questionnaire (SDQ)^19^ is a mental health screening tool widely available in population data, including in the LSAC and MCS in each of the analysed waves. Hope *et al* 6 developed and tested a measure of parent-reported MHC using eight items from the SDQ that capture the presence of key aspects of MHC, including regulating emotions and behaviour (ie, self-regulation), interacting well with peers and others (ie, social competence) and caring for others (ie, prosocial behaviour), thus making efficient use of existing data to explore MHC at a population level. Specific items included the following (online supplemental table 1), all rated as 1=not true, 2=somewhat true or 3=certainly true: (1) being considerate of other people’s feelings; (2) sharing readily with others; (3) being helpful if someone is hurt, upset or feeling ill; (4) being kind to younger children; (5) volunteering to help others; (6) being generally obedient; (7) seeing tasks through to the end; and (8) thinking things out before acting. While originally developed using data on 11-year-old children in the UK, we theorised a consistent underlying MHC construct relevant across the populations (UK, Australia) and ages (4–15 years) studied herein.

10.1136/jech-2021-216761.supp1 Supplementary data

Hope *et al* 6 analysed these items using a latent variable modelling approach to identify groups of children with different profiles of competence at one time point (11 years). In line with the purpose of this study, we took a different approach that allowed for direct comparisons of overall high MHC (which is most strongly associated with positive health and well-being outcomes^6 20^) across ages. Specifically, we summed responses to these eight items, generating total scores ranging from 8 to 24. For ease of interpretation, we used a cut point of ≥23 to indicate high MHC when estimating prevalence. This cut point had face validity, corresponding to at least seven of eight items being endorsed as ‘certainly true’ and distinguishing children with relatively high MHC from their peers, as well as providing a consistent approach across cohorts and ages. In sensitivity analyses, we also examined mean differences in the summed MHC score as a continuous variable, and patterns were consistent (online supplemental figure 1).

Our preliminary analysis added further support to the appropriateness of this MHC indicator and scoring approach for younger ages and the Australian context. A measure of MHC had been derived earlier from the teacher-reported Australian Early Development Census (AEDC^13^) and linked to records for N=2461 children in the LSAC Baby cohort.^7^ While the AEDC was not appropriate for the current research questions due to its cross-sectional design, it provided a unique validation opportunity. In the linked LSAC/AEDC sample, teacher reports on the two measures at 4–5 years were associated as expected; children scored as having high MHC on the AEDC indicator had three times the odds of high MHC on the SDQ-derived indicator (OR=3.03, 95% CI=2.31 to 3.96).

#### Sociodemographic characteristics

All sociodemographic characteristics were parent-reported in the first analysed wave (4–5 years of age), except for time invariant variables in the MCS (child sex, young maternal age at child’s birth), in which case they were taken from the first wave (9 months). These variables were categorised to create consistent indicators across the two cohorts. As the tertiary systems are more comparable than schooling levels between the two countries, we defined parent education as one or both parents having a bachelor degree (higher) versus neither parent having a bachelor’s degree (lower). Parental unemployment was defined as both parents not currently in paid work versus one or both parents working. Young maternal age was defined as 23 years or less versus more than 23 years at the child’s birth.^21^

### Analysis plan

We carried out analyses of the two cohorts separately, accounting for their respective survey designs in all analyses (see online supplemental materials for details). Item missingness and attrition over time were addressed by multiple imputation, described below. All analyses were conducted using Stata/SE V.16.1 for Windows (copyright 1985–2013; StataCorp).

#### Prevalence of MHC from 4 to 15 years of age

First, the proportion of children with high MHC (scores ≥23) was estimated at each age, in the LSAC and MCS, respectively. Proportions were estimated with 95% CIs at each survey wave, stratified by gender, and similarly the percentage difference was associated with indicators of social disadvantage (lower parental education, parental unemployment and young maternal age). As MCS participants were slightly older than LSAC participants at each time point (table 1), the proportions of children with high MHC are not directly comparable across the two cohorts.

#### Within-child change in MHC from 4 to 15 years of age

Second, change in MHC within children over time was estimated using the summed MHC scores at each age as continuous variables. The continuous MHC score was used to provide greater capacity to detect change across the full spectrum of MHC skill levels. Building on the approach of Marceau *et al*,^22^ we estimated each child’s MHC at 4–5 years and the rate of change in MHC over subsequent waves. Specifically, a linear random-effects model was fitted to individual measurements that allowed for heterogeneity between children, with four measurements nested within each child (code provided in online supplemental material). The model included a random effect for each child’s MHC at baseline (4–5 years; random intercept) and a random effect for each child’s change over time (random slope).

The random intercept and slope estimates were each split into tertiles, reflecting, first, low, medium or high baseline score and, second, decreasing, stable or increasing slope. The intersection of the intercept and slope tertiles was then used to assign each child to one of the nine possible trajectory groups. The advantage of this approach is that groups of interest are defined a priori and have a tangible relationship with the observed data, as appropriate to the descriptive aims of the study.

Finally, we examined the distribution of the trajectory groups according to gender and indicators of social disadvantage by fitting a multinomial logistic regression model between the trajectory group and the indicator.

#### Missing data

Missing data in the analysed samples ranged from 0% (eg, child’s sex collected at baseline) to 33% and 49% for MHC at 14–15 years in the LSAC and MCS, respectively (online supplemental table 2). We used multiple imputation by chained equations^23^ to impute missing data arising from attrition and item non-response within waves. The imputation model included all study variables and several auxiliary variables: parent depressive symptoms, language spoken at home (LSAC: any non-English language; MCS: mostly non-English language) and lone parent status. Data were separately imputed by child sex to preserve potential interactions between child sex and the variables within the imputation model.^24^ Fifty imputed data sets were created, and estimates were combined using Rubin’s rules.^25^

## Results

### Sociodemographic characteristics

Children in the MCS were on average slightly older than those in the LSAC at each analysed wave (table 1). There were also differences between the LSAC and MCS in demographic characteristics, reflecting their different populations and sample designs (online supplemental table 3). For example, the proportion of families with younger maternal age (23 years or less) was higher in the MCS than in the LSAC (21% and 4%, respectively), in line with national statistics around the time of sample recruitment.^26^

### Prevalence of high MHC from 4 to 15 years of age

**Figure 1:**
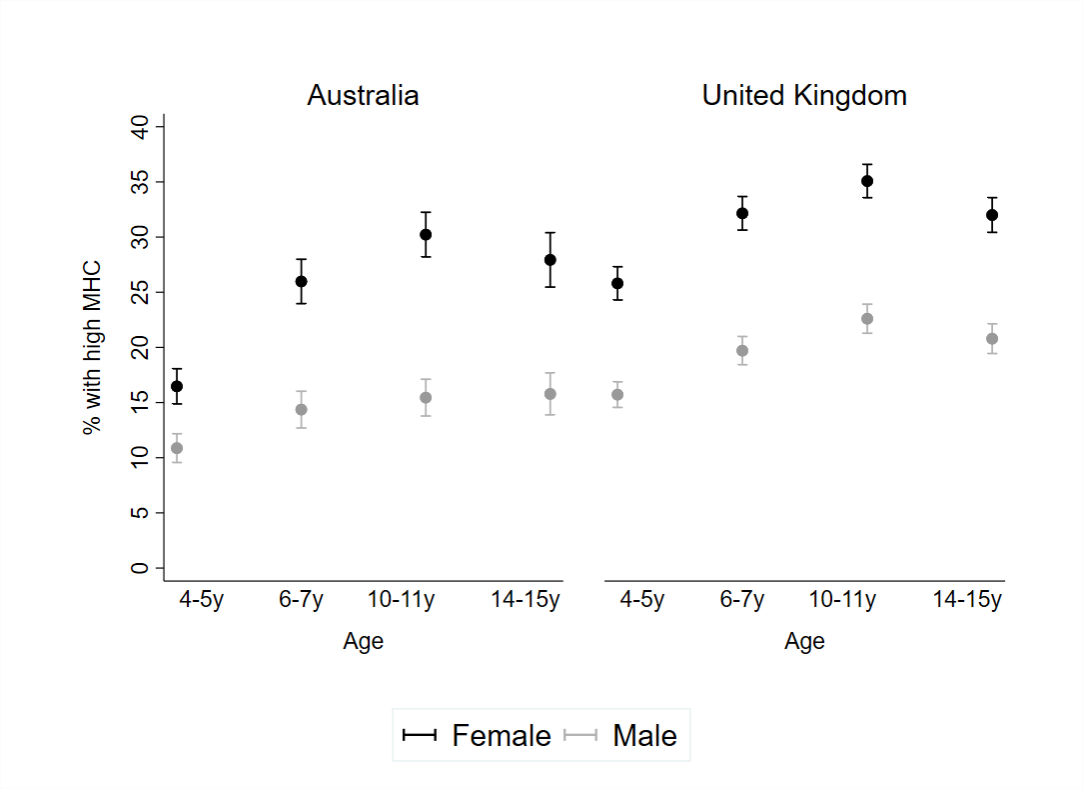
Proportion (95% CI) of children with high MHC from ages 4 to 15 years, according to country and gender. MHC, mental health competence.

In both cohorts, the proportion of children with high MHC increased between ages 4–5 years and 10–11 years, before stabilising at 14–15 years (figure 1; online supplemental table 4). The proportion of children with high MHC was substantially higher for girls compared with boys at every age. By 14–15 years, there was a 12 and 11 percentage point difference in high MHC between girls and boys in Australia and the UK, respectively.

**Figure 2:**
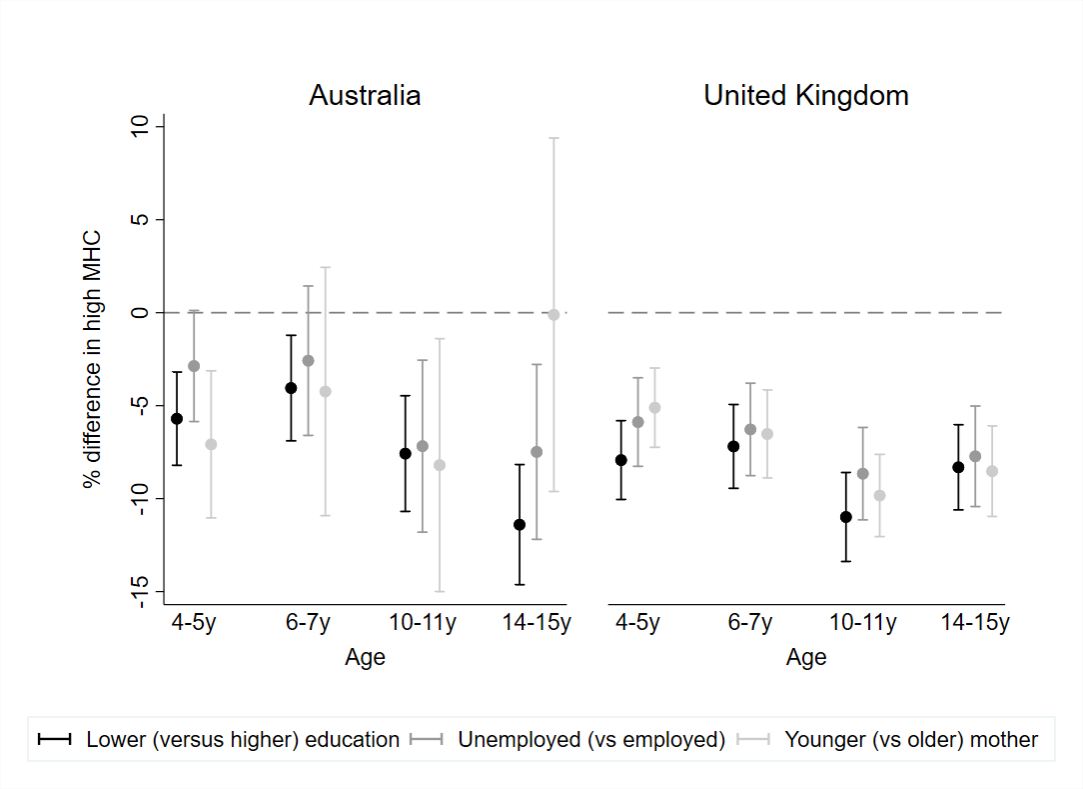
Percentage difference (95% CI) in high MHC from 4 to 15 years according to indicators of social disadvantage by country. MHC, mental health competence.

In both cohorts, the proportion of children with high MHC was consistently lower for children from more disadvantaged families compared with those from more advantaged families (figure 2; online supplemental table 5). By 14–15 years, there was an 11 and 8 percentage point difference in high MHC according to parent education, in Australia and the UK, respectively. A similar pattern was observed for parent unemployment and (in the UK, where this group was larger) young maternal age.

### Within-child change over time in MHC from 4 to 15 years of age

**Figure 3:**
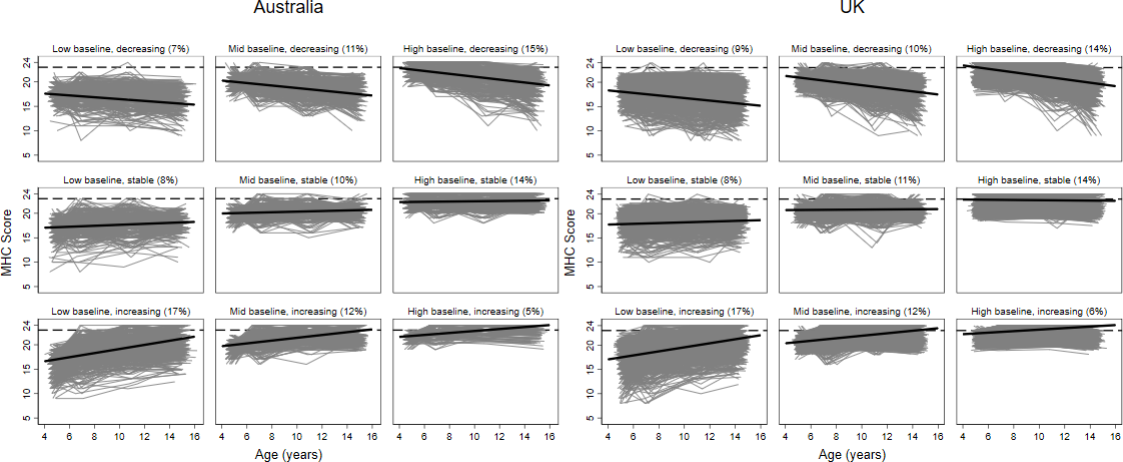
MHC trajectory groups from 4 to 15 years by country. Grey lines=each individual trajectory in the group; black solid line=estimated mean trajectory; black dashed line=cut point for high MHC (scores ≥23). MHC, mental health competence.

The most common trajectory group in both cohorts (17% in both samples) showed an initially low but increasing trajectory of MHC into adolescence (figure 3), consistent with the average pattern by age described above. However, the pathways of many children diverged from this normative course of gradual improvement in MHC. For example, 7% and 9% of children in Australia and the UK, respectively, had a low starting point and decreased further in MHC by mid-adolescence. While most children with a high baseline MHC maintained (14% in both samples) or increased in MHC (5% and 6%), a notable proportion decreased in MHC by mid-adolescence (15% and 14% in Australia and the UK, respectively).

**Figure 4:**
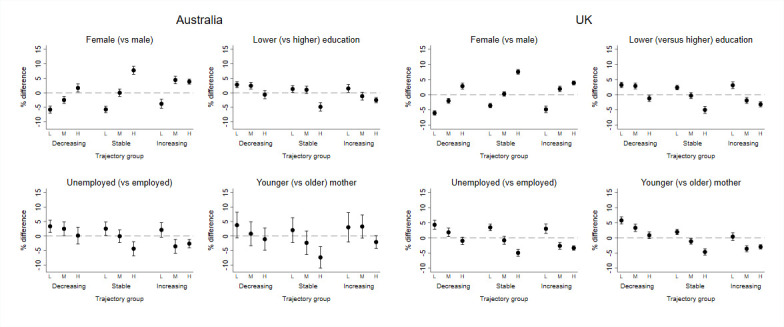
Difference in each trajectory group according to indicators of social disadvantage, gender and country. Low (L), middle (M) and high (H) refer to baseline.

Females (relative to males) and children from more advantaged backgrounds (relative to disadvantaged, for all indicators) were more likely to be classified within the high baseline and increasing trajectory group (figure 4; online supplemental table 6). Specifically, there were 4% more females than males in this trajectory group and 3% fewer children from unemployed (compared with employed) in both samples. In contrast, males and children from disadvantaged families were more likely to be classified in the low baseline and decreasing group. There were 6% more males in this group in both samples and 3% and 4% more children in this group from unemployed families, in Australia and the UK, respectively.

## Discussion

Using Australian and UK national cohort data, we examined the natural history of MHC over childhood and adolescence. Despite differences between cohort designs and countries, we found similar patterns in the development and distribution of MHC. The prevalence of high MHC showed a gradual increase as children got older, but many children deviated from this common pathway. Substantially higher proportions of females and children from more advantaged families were classified as having high MHC at each age, and trajectories that were characterised by high baseline scores and stable or increasing growth in MHC over time.

The similarity in the prevalence of high MHC by age across these national cohorts suggests that normative developmental patterns in Western countries in the 21st century entail, on average, a maturing of MHC into the adolescent years, at least as perceived by parents. Age differences in MHC were not as large as the substantial gains achieved in other developmental areas, such as cognitive skills,^27^ over this same period of the life course, however, and MHC scores stabilised by 14–15 years. The levelling off of MHC in the adolescent years may reflect unique features of this developmental period.^28^ For example, increasing autonomy brings more opportunities for missteps as young people try out new social behaviours in a wider variety of contexts, but also the potential to gain greater independent mastery of these skills.^29 30^

Beyond population patterns in the prevalence of MHC by age, there was considerable person-level variation in MHC over time. Examination of the trajectory groups suggests that even if children and young people have the same MHC starting point, they can subsequently take different pathways of MHC. For example, a notable proportion of children declined in MHC, even among those who started out with a high baseline score. This contrasts with early theoretical conceptualisations of competence as largely stable,^11^ but aligns with empirical findings for specific MHC domains such as social competence.^31^

Our findings are consistent with recent cross-sectional evidence that disadvantage is associated with lower levels of MHC.^13^ This potentially leads to a double blow for the mental health of disadvantaged children, given they are also at higher risk of experiencing mental disorder.^32^ The indicators of disadvantage examined here reflect access to resources that allow families to maximise opportunities to promote their children’s MHC, as well as avoid exposure to risks and adversities that undermine healthy development.^33^ For example, families living in more advantaged neighbourhoods have better access to high-quality early childhood education and care settings that support the social and emotional aspects of development (eg, through responsive child–teacher interactions).^34^

Sex differences in the prevalence of MHC were also observed, consistent with previous research.^5 13^ This is likely to be the consequence of a range of factors. For example, early socialisation processes place greater value on prosocial and caring behaviours for girls compared with boys.^35^ Boys also have higher levels of special healthcare needs compared with girls,^36^ and their social and emotional development appears to be more heavily impacted by adverse environmental conditions.^37 38^ While there were differences in prevalence by sex, it is notable that age patterns over time were similar across genders.

### Strengths and limitations

As with all aspects of mental health,^39^ the measurement of MHC is complex and requires a variety of approaches able to meet different study objectives. We used an approach that was efficient and appropriate to the epidemiological aims of the analyses because of the widespread inclusion of the SDQ (from which our MHC items were drawn) in population cohorts. Scoring, using a simple sum of responses, was also appropriate to the study aims, reflecting high levels of overall skill. In future, it would be valuable to develop brief, purpose-derived measures of MHC that could be feasibly included in population surveys, capture a wider spectrum of MHC skill levels, have cut points corresponding to meaningful thresholds and are culturally appropriate and validated across a range of population groups (eg, by gender and for children and adolescents from ethnic minority and from Indigenous backgrounds).

We focused on parent reports of their child’s MHC, as these were collected at every data collection wave from early childhood through to adolescence in both cohorts. Theoretical approaches emphasise that competence is relative to expectations of a child’s behaviour at a given age.^1^ Parents are therefore likely to calibrate their ratings on the basis of both the child’s demonstrated skills and their own expectations of behaviour, as observed primarily in the family environment. In future research, triangulation with self-reported and teacher-reported MHC, and with observations of skills occurring in daily situations, would be valuable.

In measuring social adversity, we examined indicators at one time point only (baseline) because factors such as parent education and occupation tend to remain stable over time.^40^ However, families’ circumstances can change, and the investigation of temporal patterns of exposure would be valuable. Furthermore, we examined adversity according to parent education, occupation and young maternal age. While beyond the scope of this article, investigating how such factors interact, as well as other sources of marginalisation and social stratification such as ethnicity, Indigenous status and disability, is also important. For example, in both Australia and the UK, young people from ethnic minority backgrounds can experience racial discrimination and marginalisation within the systems and institutions of society, contributing to mental health inequities.^41^

Families facing extreme adversity, such as those experiencing homelessness, are likely under-represented due to difficulties accessing and engaging these families. In addition, attrition has occurred in the LSAC and MCS, where the availability of MHC assessments by 15 years reflected 10 years and 15 years of retention, respectively, and this has been differentially higher for the most disadvantaged. We have used multiple imputation to reduce the potential for differential attrition to impact study findings. Nevertheless, prevalence estimates may overestimate the proportions of children with high MHC in both populations.

### Implications and future research

The current findings have implications for the timing of interventions to improve MHC. The gradual maturation of skills observed is consistent with the notion that ‘skill begets skill’,^42^ whereby children with strong early skills are able to take advantage of environmental and learning opportunities to develop increasingly sophisticated capacities. Opportunities to promote a strong foundation of the skills that underlie MHC in the early years, such as through embedding social and emotional learning programmes in early childhood education and care settings,^43^ should be further explored. These results also highlight the importance of continued focus on MHC into adolescence because high MHC in the early years does not ensure continued high MHC, and conversely, low initial MHC does not preclude MHC increasing into adolescence.

The potential to reduce differences in MHC by socioeconomic circumstances and gender has typically been only peripherally examined in intervention research, and not systematically reported for socioeconomic circumstances (eg, only available for around half of the studies meta-analysed by Taylor *et al* 9). Our findings suggest that sociodemographic disparities should be more central to the design and evaluation of interventions. This includes stating an explicit aim of reducing disparities and directly testing whether this aim has been achieved. Doing so is likely to require additional, more intensive interventions for particular groups who would benefit most,^44^ including those children from disadvantaged backgrounds and boys.

Finally, continued research is needed to examine environmental and individual factors that drive stability and change in MHC, including for specific groups such as those from socially disadvantaged backgrounds. For example, the method adopted here to assign individuals to trajectory groups can be used to examine early, and potentially intervenable, predictors of trajectory group membership. Exploring the role of factors that may differentiate those groups with a similar starting point who diverged in their subsequent MHC pathways will be of particular importance.

## Conclusions

The study of positive aspects of mental health, as opposed to disease, has traditionally been neglected in epidemiological research.^45^ Yet competence across core psychosocial capabilities such as caring for others, engaging in healthy relationships and managing emotions is both intrinsically valued and predicts a broad range of psychosocial, health and educational outcomes.^2 3 8^ We present a developmental picture of the natural history of MHC from childhood through adolescence, finding similarities between Australian and UK contexts. Our findings suggest that efforts to promote MHC in childhood and adolescence require a focus on building competence in the early years, sustaining those skills throughout childhood and into adolescence, and reducing disparities in MHC for boys and children from disadvantaged backgrounds.

**What is already known on this subject**

- The study of positive aspects of mental health, as opposed to disease, has traditionally been neglected in epidemiological research. Mental health competence involves skills such as being able to regulate emotions, interact well with peers and care for others. These skills are both intrinsically valued and predict a broad range of psychosocial, health and educational outcomes. Interventions have shown the potential to achieve sustained improvements in key competence skill areas.

**What this study adds**

- Findings reveal that, in general, mental health competence steadily increases between childhood and adolescence. However, some children experience other pathways, such as declining competence during this period, and this was more common for boys and children from socially disadvantaged backgrounds. Approaches to promoting competence should focus on sustained efforts to build competence from early childhood to adolescence and on reducing sociodemographic disparities.

## Data Availability

Data used in this manuscript are available upon reasonable request from the Longitudinal Study of Australian Children (https://growingupinaustralia.gov.au/) and the Millennium Cohort Study (https://cls.ucl.ac.uk/cls-studies/millennium-cohort-study/). In partnership with the Australian Data Archive, the National Centre for Longitudinal Data (NCLD) facilitates access to LSAC data using Dataverse (https://dataverse.ada.edu.au/dataverse.xhtml?alias=lsac). Millennium Cohort Study data are held by the UK Data Service for all the sweeps (first survey https://doi.org/10.5255/UKDA-SN-4683-1; second survey http://doi.org/10.5255/UKDA-SN-5350-3; third survey http://doi.org/10.5255/UKDA-SN-5795-3; fourth survey http://doi.org/10.5255/UKDA-SN-6411-6; fifth survey http://doi.org/10.5255/UKDA-SN-7464-2; sixth survey http://doi.org/10.5255/UKDA-SN-8156-2).
