# Supplementary material for: Natural history of mental health competence from childhood to adolescence
Source: J Epidemiol Community Health. 2021 Aug 16;76(2):133–9. doi: 10.1136/jech-2021-216761 (PMC8762025; doi:10.1136/jech-2021-216761)
Supplement: Supplementary data [file jech-2021-216761supp001.pdf]

Supplementary material

Supplementary Table 1. Parent reported Strengths and Difficulties Questionnaire (SDQ) items used to measure MHC in the LSAC and MCS.

SDQ subscale	Item
Prosocial behaviours scale	Considerate of other people's feelings
	Shares readily with others
	Helpful if someone is hurt, upset or feeling ill
	Kind to younger children
	Often volunteers to help others (parents, teachers, other children)
Conduct problems	Generally obedient, usually does what adults request
Hyperactivity	Sees tasks through to the end
	Thinks things out before acting

Note. Parents reported on a 3-point scale, 1=not true; 2=somewhat true; 3=certainly true. There were minor wording changes to items between child and youth waves.

Supplementary Table 2. Number and proportion (%) of participants with missing data on study variables in the LSAC (N=4983) and MCS (N=18,296).

Variable	LSAC n(%)	MCS n(%)
Mental health competence (SDQ)		
4-5 years	20 (0.4%)	8039 (43.9%)
6-7 years	683 (13.7%)	8069 (44.1%)
10-11 years	869 (17.4%)	6958 (38.0%)
14-15 years	1617 (32.5%)	8865 (48.5%)
Sociodemographic characteristics (4-5 years)		
Child’s sex	0 (0%)	0 (0%)
Parental education	63 (1.3%)	3043 (16.6%)
Parent unemployment	17 (0.3%)	3835 (21.0%)
Young maternal age at child’s birth	2 (0.0%)	58 (0.3%)

Supplementary Table 3. Sociodemographic characteristics of participants in the LSAC (N=4,983) and MCS (N=18,296).

	**LSAC (N = 4,983)**			**MCS (N = 18,296)**		
	%	95% CI		%	95% CI	
Child's sex						
Male	51.2	49.8	52.7	51.4	50.5	52.2
Female	48.8	47.3	50.2	48.6	47.8	49.5
Parental education						
Higher (degree level)	27.2	24.7	29.6	28.7	26.4	31.0
Lower (below degree)	72.8	70.4	75.3	71.3	69.0	73.6
Parent unemployment						
Working parent	87.0	85.7	88.3	84.7	83.7	85.8
Neither parent in paid employment	13.0	11.7	14.3	15.3	14.2	16.3
Young maternal age at child’s birth						
Over 23 years of age	96.0	95.3	96.8	79.1	77.8	80.5
Equal to or less than 23 years	4.0	3.2	4.7	20.9	19.5	22.2
Lone parent						
Partnered	84.9	83.7	86.2	81.5	80.5	82.6
Lone parent	15.1	13.8	16.3	18.5	17.4	19.5
Language background						
English	84.6	82.5	86.7	97.2	96.6	97.9
Non-English	15.4	13.3	17.5	2.8	2.1	3.4

Supplementary Table 4. Proportion (with 95% CI) of children with high MHC from 4-5 to 14-15 years according to country and gender. Estimates corresponding to Figure 1.

**Age**	**Australia**									**UK**								
	**Male**			**Females**			**All**			**Male**			**Females**			**All**		
	**%**	**95% CI**		**%**	**95% CI**		**%**	**95% CI**		**%**	**95% CI**		**%**	**95% CI**		**%**	**95% CI**	
4-5 yrs	10.87	9.57	12.17	16.47	14.87	18.06	13.60	12.57	14.63	15.72	14.56	16.88	25.80	24.30	27.31	20.62	19.65	21.60
6-7 yrs	14.36	12.70	16.03	25.98	23.96	27.99	20.03	18.68	21.37	19.71	18.42	20.99	32.15	30.63	33.67	25.76	24.72	26.80
10-11 yrs	15.45	13.78	17.12	30.23	28.20	32.25	22.66	21.28	24.03	22.60	21.28	23.92	35.08	33.57	36.59	28.67	27.60	29.74
14-15 yrs	15.79	13.88	17.69	27.93	25.47	30.40	21.71	20.09	23.33	20.79	19.44	22.14	32.00	30.43	33.57	26.24	25.12	27.37

Supplementary Table 5. Proportion of children with high mental health competence from 4-5 to 14-15 years according to country and indicators of social disadvantage. Estimates corresponding to Figure 2.

**Group comparison**	**Australia**											
	**4-5 years**			**6-7 years**			**10-11 years**			**14-15 years**		
	**% diff**	**95% CI**		**% diff**	**95% CI**		**% diff**	**95% CI**		**% diff**	**95% CI**	
Lower (versus higher) education	-5.7	-8.21	-3.19	-4.05	-6.89	-1.22	-7.58	-10.69	-4.47	-11.4	-14.63	-8.17
Unemployed (vs employed)	-2.87	-5.86	0.12	-2.58	-6.6	1.44	-7.18	-11.8	-2.55	-7.49	-12.19	-2.78
Younger (vs older) mother	-7.08	-11.04	-3.13	-4.24	-10.92	2.44	-8.2	-15	-1.4	-0.11	-9.62	9.39
**Group comparison**	**UK**											
	**4-5 years**			**6-7 years**			**10-11 years**			**14-15 years**		
	**% diff**	**95% CI**		**% diff**	**95% CI**		**% diff**	**95% CI**		**% diff**	**95% CI**	
Lower (versus higher) education	-7.93	-10.05	-5.81	-7.19	-9.44	-4.94	-10.99	-13.38	-8.6	-8.31	-10.6	-6.03
Unemployed (vs employed)	-5.88	-8.26	-3.5	-6.28	-8.77	-3.79	-8.66	-11.14	-6.17	-7.72	-10.43	-5.02
Younger (vs older) mother	-5.11	-7.24	-2.98	-6.52	-8.89	-4.16	-9.83	-12.04	-7.62	-8.53	-10.96	-6.09

Supplementary Table 6. Proportion of children in each trajectory group according to country and indicators of social disadvantage. Estimates corresponding to Figure 4.

**Trajectory group**	**Australia**											
	**Female (vs male)**			**Lower (vs higher) education**			**Unemployed (vs employed)**			**Younger (vs older) mother**		
	**% diff**	**95% CI**		**% diff**	**95% CI**		**% diff**	**95% CI**		**% diff**	**95% CI**	
Low baseline, decreasing	-5.78	-7.00	-4.57	2.81	1.77	3.85	3.35	1.24	5.45	3.76	-0.67	8.20
Mid baseline, decreasing	-2.47	-3.66	-1.27	2.45	1.29	3.60	2.49	0.11	4.87	0.80	-3.32	4.92
High baseline, decreasing	1.68	0.29	3.07	-0.63	-2.04	0.77	0.14	-2.75	3.04	-1.09	-4.91	2.74
Low baseline, stable	-5.72	-6.84	-4.61	1.32	0.16	2.48	2.53	0.23	4.83	2.03	-2.25	6.31
Mid baseline, stable	0.02	-1.28	1.31	1.05	-0.21	2.31	-0.06	-2.27	2.15	-2.34	-6.42	1.74
High baseline, stable	7.77	6.37	9.17	-4.83	-6.27	-3.39	-4.37	-6.80	-1.95	-7.36	-11.08	-3.65
Low baseline, increasing	-3.78	-5.32	-2.23	1.50	0.08	2.92	2.11	-0.43	4.66	3.00	-2.07	8.07
Mid baseline, increasing	4.43	3.13	5.74	-1.15	-2.50	0.20	-3.57	-5.95	-1.18	3.28	-0.73	7.28
High baseline, increasing	3.85	3.00	4.71	-2.51	-3.41	-1.61	-2.63	-4.05	-1.20	-2.08	-4.28	0.13
**Trajectory group**	**UK**											
	**Female (vs male)**			**Lower (vs higher) education**			**Unemployed (vs employed)**			**Younger (vs older) mother**		
	**% diff**	**95% CI**		**% diff**	**95% CI**		**% diff**	**95% CI**		**% diff**	**95% CI**	
Low baseline, decreasing	-6.03	-6.75	-5.31	3.23	2.36	4.11	4.34	2.86	5.83	5.77	4.59	6.96
Mid baseline, decreasing	-2.03	-2.84	-1.22	2.89	1.92	3.86	1.85	0.45	3.25	3.32	2.11	4.53
High baseline, decreasing	2.84	1.84	3.84	-1.22	-2.23	-0.21	-0.97	-2.19	0.25	0.87	-0.25	1.99
Low baseline, stable	-3.58	-4.29	-2.88	2.36	1.65	3.08	3.46	2.38	4.54	1.94	1.07	2.81
Mid baseline, stable	0.26	-0.47	0.99	-0.30	-1.28	0.68	-0.80	-2.12	0.51	-1.14	-2.08	-0.19
High baseline, stable	7.56	6.79	8.33	-5.02	-6.18	-3.86	-4.94	-6.09	-3.78	-4.64	-5.68	-3.61
Low baseline, increasing	-4.85	-5.93	-3.77	3.12	2.02	4.23	3.04	1.50	4.58	0.42	-0.85	1.68
Mid baseline, increasing	1.93	1.02	2.83	-1.89	-2.87	-0.92	-2.63	-3.77	-1.50	-3.59	-4.59	-2.58
High baseline, increasing	3.91	3.26	4.55	-3.18	-4.00	-2.35	-3.34	-4.08	-2.60	-2.96	-3.66	-2.25

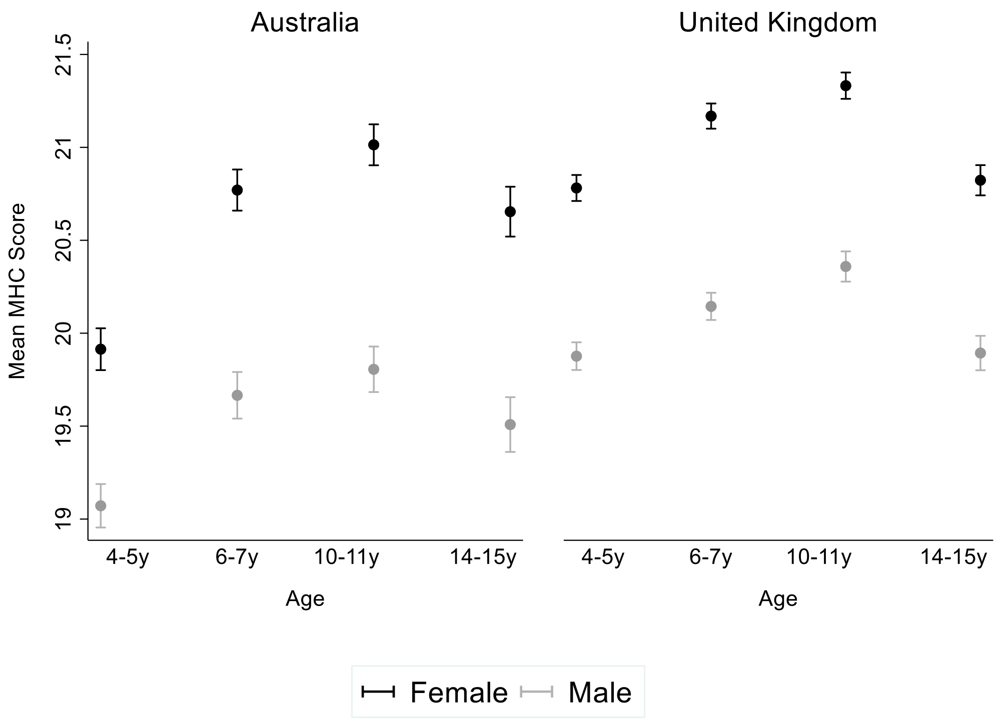

Supplementary Figure 1. Mean (95% CI) continuous MHC score from ages 4 to 15 years, according to country and gender.

Approach to accounting for survey design in each cohort

Proportions of children with high MHC from 4-15 years of age

To account for stratification in the survey design, clustering within the primary sampling unit, and survey weights, the svyset stata command was used. For example, this was implemented as mi svyset [pweight=zweight] zpsun, strata(zstrn) singleunit(scaled), where the primary sampling unit (zpsun) refers to the original variables SPTN00 and pcodes in the MCS and LSAC respectively; strata (zstrn) to PTTYPE2 and stratum; and the wave 1 weighting variable (zweight) to AOVWT2 and cweight. We note that the Wave 1 weighting variable was applicable for estimates at all waves included as subsequent sample attrition was accounted for using multiple imputation. Estimates were made on a wave specific basis thus no additional adjustment for repeated measurements was required.[1]

Approach to deriving trajectory groups to explore within individual change over time in MHC from 4-15 years

Additional notes on method

Cut points at the 33rd and 66th percentile of the random slopes do not impose a direction or magnitude on the slope. For example, all tertiles may comprise of increasing slopes. However, in these data, the cut points can be interpreted as stated.

Assignment of each individual to one of the nine pre-specified trajectory groups, and estimates of the distribution of the nine trajectory groups across gender and the indicators of social disadvantage, were performed for each imputation, with results combined using Rubin's rules.

To estimate the mean intercept and change over time, and corresponding confidence interval, in each of the nine trajectory groups, we fit a random effects model between age and MHC score. Change over time was assumed to follow a linear relationship with age.

Stata code for trajectory models

Code is provided below to illustrate the trajectory modelling approach and inform approaches of other researchers interested; it should not be applied without careful consideration of and validation within different data and contexts. For brevity, data cleaning and imputation steps are not presented.

```
********************************************************************************
* data dictionary
********************************************************************************

* ahedpn_hi:                Education level present in the household
* ahempd:                   Neither parent in paid employment
* alonpn:                   Lone parent
* zagepn:                   Mothers age at child's birth
* zsexpn:                   Child's sex
* zcounn:                   Country
* mhcpd:                    Mental health competency score
* agep_childage:            Child's age at assessment
* zweight:                  Wave 1 population weight
* ID:                       Unique identifier for each child
* wave:                     Wave identifier

********************************************************************************
* DO FILE 1 - classify trajectories
********************************************************************************
* Author:       Sarah Arnup
* Purpose:      This do file classifies each imputed trajectory into one of
*                      nine groups based on intercept and slope tertile,
```

```
*                      as obtained from a mixed model, with random effect
*                      for intecept and slope.
* Date:        27 Nov 2019
* Edited:      22 Jun 2020
********************************************************************************

version 16.0
clear all

*** open data
*** data in long format

* manage data
********************************************************************************
* change mi format to flong for mixed modelling
mi convert flong, clear

* trajectory modelling
********************************************************************************
* set up variables needed to extract trajectory groups
forval y=1/9 {
               gen b_slope`y' = .
               gen se`y' = .
               gen intercept`y' = .

               foreach var of varlist ahedpn_hi ahempd alonpn zagepn zsexpn zcounn {
                      gen mean_`var'`y' = .
                      gen var_`var'`y' = .
               }
}

gen baseline = .
gen slope = .
gen groups = .

* obtain number of imputations
mi query
local M = r(M)

* iterate through imputations
forvalues i = 1/`M' {

               * for each imputation, obtain random effect and slope for each individual
               * include pweights at the level of the individual, not observation
               mi xeq `i': meglm mhcpd agep_childage || ID: wave, covariance(unstructured)
pweight(zweight)

               * obtain estimates of random effects
               predict b* if _mi_m==`i', reffects

               * obtain 33rd and 66th centile of random intercept
               centile b1 if _mi_m==`i', centile(33)
               local p33 = r(c_1)
               centile b1 if _mi_m==`i', centile(66)
               local p66 = r(c_1)

               * create baseline categories for intercept based on tertile
               gen bl = 1 if b1 < `p33' & _mi_m==`i'
               replace bl = 2 if b1 < `p66' & bl==. & _mi_m==`i'
               replace bl = 3 if b1 < . & bl==. & _mi_m==`i'
               replace baseline = bl if _mi_m==`i'

               * obtain 33rd and 66th centile of random slope
               centile b2 if _mi_m==`i', centile(33)
               local p33 = r(c_1)
               centile b2 if _mi_m==`i', centile(66)
               local p66 = r(c_1)

               * create slope categories for slope based on tertile
               gen s = 1 if b2 < `p33' & _mi_m==`i'
               replace s = 2 if b2 < `p66' & s==. & _mi_m==`i'
               replace s = 3 if b2 < . & s==. & _mi_m==`i'
               replace slope = s if _mi_m==`i'

               * create 9 groupings of baseline and slope tertiles
               egen g = group(baseline slope) if _mi_m==`i'
               replace groups = g if _mi_m==`i'
```

```
                * drop varibles needed for next iteration
                drop b? bl s g
}

compress
save "imputed trajectories_re_groups", replace

exit

********************************************************************************
* DO FILE 2 - obtain estimate of slope and intercept for each trajectory group
********************************************************************************

********************************************************************************
* Author:       Sarah Arnup
* Purpose:      Obtain estimate of slope and intercept for each group, with
*                       variance obtained using Rubin's rules.
* Date:         27 Nov 2019
* Edited:       22 June 2020
********************************************************************************

* Notes:
*       Rubin's Rules: V_T = V_W + (1+(1/m))V_B
*       V_W = 1/m sum (Vi)
*       V_B = (1/(m-1)) sum (Yi - Ybar)^2
*       df = (m-1) ( 1+ V_W / ((1+(1/m))V_B) )^2

*       Student's T-distribution with v degrees of freedom.
*       CI Ybar +- t_df, (1-alpha/2) sqrt(V_T)
*       increase in variance due to missing data
*               sqrt(V_T/V_W) - 1

********************************************************************************

version 16.0
clear all

* open data
use "imputed trajectories_re_groups"

* we wish to obtain an estimate of the slope in each group
* fit linear regression to each of nine groups
********************************************************************************

* obtain number of imputations
mi query
local M = r(M)

* iterate through imputations
forvalues i = 1/`M' {

                * iterate through each of the 9 groups
                forval y=1/9 {

                        * fit a linear regression, accounting for repeated measurements within
individual
                        regress mhcpd agep_childage if groups == `y' & _mi_m==`i', vce(cluster
ID) noheader

                        * extract intercept, beta and se for each regression
                        replace b_slope`y' = _b[agep_childage] if _mi_m==`i' & groups == `y'

                        replace se`y' = _se[agep_childage] if _mi_m==`i' & groups == `y'
                        replace intercept`y' = _b[_cons] if _mi_m==`i' & groups == `y'
                }
}

* Obtain variances using Rubins Rules for estimate of slope in each group
********************************************************************************
* "bysort ID wave:" will sum each observation over the 50 runs
* run number is given by _mi_m

* drop observed data
drop if _mi_m == 0

* create indicators
```

```
gen one = 1
bysort ID wave: egen check = total(one) // this should total the number of imputations, i.e.
50

* create mean of mhc over all imputations
bysort ID wave: egen y_imputed = mean(mhcpd) // needed for figure, isn't used further

* for each imputation (_mi_m) the regression parameters will be constant within each group
* we want to create a complete dataset with regression parameters for each group
forval y=1/9 {
        bysort _mi_m (b_slope`y'): replace b_slope`y' = b_slope`y'[1]
        bysort _mi_m (intercept`y'): replace intercept`y' = intercept`y'[1]
        bysort _mi_m (se`y'): replace se`y' = se`y'[1]
}

* create the within and between and total variance, and d.f.
forval y=1/9 {

        * create the variance of the estimate of slope
        gen var`y' = (se`y')^2

        * this is V_W: within imputation variance for each observation
        bysort ID wave: egen V_W`y'= mean(var`y')

        * create mean regression parameters for each observation for each group
        bysort ID wave: egen mean_slope`y' = mean(b_slope`y')
        bysort ID wave: egen mean_intercept`y' = mean(intercept`y') // this isn't used

        * create difference between mean and observation for slope for each observation
        gen between`y' = (b_slope`y' - mean_slope`y')^2

        * calculate V_B: between impuation variance
        bysort ID wave: egen sum_between`y' = total(between`y')
        gen V_B`y' = sum_between`y' / (check-1)

        * calcuate total variance and dfs
        gen V_T`y' = V_W`y' + (1+(1/check))*V_B`y'
        gen df`y' = (check-1) * ( 1 + V_W`y' / ( ( 1+(1/check) )* V_B`y' ) )^2
}


compress

save "imputed trajectories_trajectories", replace
exit


********************************************************************************
* DO FILE 3 - Obtain estimate of the probability of being in each group,
*                              given socio-demographic factor
********************************************************************************

*********************************************************************************
* Author:       Sarah Arnup
* Purpose:      Obtain estimate of the probability of being in each group,
*                       given socio-demographic factor, with variance obtained using
*                       Rubin's rules as per do-file 2.
* Date:         27 Nov 2019
* Edited:       22 June 2020
********************************************************************************

version 16.0
clear all

* open data
use "imputed trajectories_re_groups"

* tidy up data
********************************************************************************
* drop observed data
drop if _mi_m ==0

* drop unneeded variables
drop mhcpd mhcpd_hi
drop *slope* se* intercept* baseline slope agep_childage
compress
```

```
* create some indicator variables
gen one = 1
bysort ID wave: egen check = total(one)

* estimate probabilities for each group using multinomial logistic regression
* and margins
********************************************************************************

* obtain number of imputations
mi query
local M = r(M)

* estimate proportion
forvalues i = 1/50 {

               * repeat for each imputation run
               preserve
                       keep if _mi_m==`i'

                       * repeat for each socio-demographic factor
                       foreach var of varlist ahedpn_hi ahempd alonpn zagepn zsexpn {

                               * run multinomial logistic regression
                               mlogit groups i.`var'

                               *run margins to estimate effect of factor on probability
                               margins, dydx(`var')
                               * extract table of estimates
                               mat table1 = r(table)

                               * run margins to obtain probabilities for each level of factor
in each group
                               margins `var', post
                               * extract table of estimates
                               mat table2 = r(table)

                               * extract estimates
                               forval y = 1/9 {

                                       * extract effect estimate and variance
                                       local a = 9 + `y'
                                       replace mean_`var'`y' = table1[1,`a']
                                       replace var_`var'`y' = table1[2,`a']
                                       replace var_`var'`y' = (var_`var'`y')^2

                                       * extract mean and variance of probability for each
group
                                       local a0 = (2*`y') - 1
                                       local a1 = 2*`y'
                                       gen mean_`var'0_`y' = table2[1,`a0']
                                       gen mean_`var'1_`y' = table2[1,`a1']
                                       gen var_`var'0_`y' = table2[2,`a0']
                                       gen var_`var'1_`y' = table2[2,`a1']
                                       replace var_`var'0_`y' = (var_`var'0_`y')^2
                                       replace var_`var'1_`y' = (var_`var'1_`y')^2
                               }
                       }

                       compress
                       * save separate data file for each imputation
                       save "imputed_trajectories_proportions_`i'",replace
               restore

               }

clear all

* append all imputation data files together
********************************************************************************
use "imputed_trajectories_proportions_1"
       forval i=2/50 {
               append using "imputed_trajectories_proportions_`i'"
       }
       save "imputed_trajectories_proportions_1_50",replace
}
```

```
* calculate estimate and variance for each socio-demographic factor
********************************************************************************
* (follows the same methodology as do-file 2)

*** REPEAT for each of the indicators: ahedpn_hi ahempd alonpn zagepn zsexpn zcounn

* open appended data
use "imputed_trajectories_proportions_1_50", clear all

* unset data
mi unset, asis

* calculate estimate and variance as per do-file 2
foreach var of varlist ahedpn_hi {
forval y=1/9 {
	noi di `y'
	bysort _mi_m (mean_`var'`y'): replace mean_`var'`y' = mean_`var'`y'[1]
	bysort _mi_m (var_`var'`y'): replace var_`var'`y' = var_`var'`y'[1]
	bysort ID wave: egen mean_mean_`var'`y'= mean(mean_`var'`y')
	bysort ID wave: egen mean_var_`var'`y'= mean(var_`var'`y')
	gen between_`var'`y' = (mean_`var'`y' - mean_mean_`var'`y')^2
	bysort ID wave: egen sum_between_`var'`y' = total(between_`var'`y')
	gen V_B`var'`y' = sum_between_`var'`y' / (check -1)
	gen V_T`var'`y' = mean_var_`var'`y' + (1+(1/check))*V_B`var'`y'
	gen df`var'`y' = (check-1) * ( 1 + mean_var_`var'`y' / ( ( 1+(1/check) )* V_B`var'`y'
) )^2

	forval x=0/1 {
		bysort _mi_m (mean_`var'`x'_`y'): replace mean_`var'`x'_`y' =
mean_`var'`x'_`y'[1]
		bysort _mi_m (var_`var'`x'_`y'): replace var_`var'`x'_`y' = var_`var'`x'_`y'[1]
		bysort ID wave: egen mean_mean_`var'`x'_`y'= mean(mean_`var'`x'_`y')
		bysort ID wave: egen mean_var_`var'`x'_`y'= mean(var_`var'`x'_`y')
		gen between_`var'`x'_`y' = (mean_`var'`x'_`y' - mean_mean_`var'`x'_`y')^2
		bysort ID wave: egen sum_between_`var'`x'_`y' = total(between_`var'`x'_`y')
		gen V_B`var'`x'_`y' = sum_between_`var'`x'_`y' / (check -1)
		gen V_T`var'`x'_`y' = mean_var_`var'`x'_`y' + (1+(1/check))*V_B`var'`x'_`y'
		gen df`var'`x'_`y' = (check-1) * ( 1 + mean_var_`var'`x'_`y' / ( ( 1+(1/check)
)* V_B`var'`x'_`y' ) )^2
	}
}
}

* tidy up data
********************************************************************************
keep in 1
keep df* V_T* mean_ahedpn_hi*
gen name = 1

reshape long dfahedpn_hi dfahedpn_hi0_ dfahedpn_hi1_ V_Tahedpn_hi V_Tahedpn_hi0_
V_Tahedpn_hi1_ mean_ahedpn_hi mean_ahedpn_hi0_ mean_ahedpn_hi1_, j(wave) i(name)

* calculate confidence intervals
********************************************************************************
rename mean_ahedpn_hi mean
rename V_Tahedpn_hi V_T
rename dfahedpn_hi df
rename mean_ahedpn_hi0 mean0
rename V_Tahedpn_hi0 V_T0
rename dfahedpn_hi0 df0
rename mean_ahedpn_hi1 mean1
rename V_Tahedpn_hi1 V_T1
rename dfahedpn_hi1 df1

gen ci = invt(df,0.975) * sqrt(V_T)
gen ci_l = mean - ci
gen ci_u = mean + ci

gen ci0 = invt(df0,0.975) * sqrt(V_T0)
gen ci_l0 = mean0 - ci0
gen ci_u0 = mean0 + ci0

gen ci1 = invt(df1,0.975) * sqrt(V_T1)
gen ci_l1 = mean1 - ci1
gen ci_u1 = mean1 + ci1

* label trajectories
```

```
rename wave trajectory
label define traj 1 "Low baseline, decreasing" ///
 2 "Mid baseline, decreasing" ///
 3 "High baseline, decreasing" ///
 4 "Low baseline, stable" ///
 5 "Mid baseline, stable" ///
 6 "High baseline, stable" ///
 7 "Low baseline, increasing" ///
 8 "Mid baseline, increasing" ///
 9 "High baseline, increasing"
label values trajectory traj

order name trajectory mean ci_l ci_u mean0 ci_l0 ci_u0 mean1 ci_l1 ci_u1

save "MHCproportions_ahedpn_hi", replace

exit


******************************************************************************
* DO FILE 4 - construct trajectories for each group based on intercept and slope
******************************************************************************

******************************************************************************
* formula for line
* y1 = mean_b_slope1 * agep_childage + mean_intercept1
* 95% CI t_df1, 1-alpha/2 * sqrt(V_T1)
* 95% CI invt(df1,0.975) * sqrt(V_T1)

version 16.0
clear all

* open data
use "imputed_trajectories"

* keep first imputation as example 'raw' trajectories
keep if _mi_m==1

* create some dummy IDs for mean trajectory per group
count
local temp = _N + 2
set obs `temp'
replace ID = 1 if _n==_N-1
replace ID = 2 if _n==_N

* replicate one for each of 9 groups
gen replicate = 1 if ID ==1|ID==2
expand 9 if replicate==1
gsort -ID
count
replace groups = (_N - _n) + 1 in -9/-1
replace groups = (_N - _n) - 8 in -18/-10

* create variables needed for each line
forval y=1/9 {
        gen y`y'=.
        gen ci`y' = .
        gen ci_l`y' = .
        gen ci_u`y' = .
}

* set age at 3 for first point
replace agep_childage = 4*12 if ID==1
forval y=1/9 {
        replace y`y' = mean_slope`y'[1] * agep_childage + mean_intercept`y'[1] if ID==1 &
groups==`y'
}

* set age at 16 for second point
replace agep_childage = 16*12 if ID==2
forval y=1/9 {
        replace y`y' = mean_slope`y'[1] * agep_childage + mean_intercept`y'[1] if ID==2 &
groups==`y'
}

* calculate CI (very narrow)
forval y=1/9 {
```

```
        replace ci`y' = invt(df`y'[1],0.975) * sqrt(V_T`y'[1]) if (ID==1 | ID==2) &
groups==`y'
        replace ci_l`y' = y`y' - ci`y' if (ID==1 | ID==2) & groups==`y'
        replace ci_u`y' = y`y' + ci`y' if (ID==1 | ID==2) & groups==`y'
}

* y_imputed contains mean trajectory per groupings

exit
```

Supplemental references

1 Spittal MJ, Carlin JB, Currier D*, et al.* The Australian Longitudinal Study on Male Health sampling design and survey weighting: Implications for analysis and interpretation of clustered data. *BMC Public Health* 2016;**16**:1062.
